# Jagged1 protein processing in the developing mammalian lens

**DOI:** 10.1242/bio.041095

**Published:** 2019-03-19

**Authors:** Mina Azimi, Nadean L. Brown

**Affiliations:** Department of Cell Biology & Human Anatomy, University of California, 1 Shields Avenue, Davis, CA 95616, USA

**Keywords:** Jagged1, Notch signaling, Presenilin, Gamma secretase (γ-secretase), ADAM protease, Lens development

## Abstract

Notch signaling regulates a multitude of cellular processes. During ocular lens development this pathway is required for lens progenitor growth, differentiation and maintenance of the transition zone. After ligand-receptor binding, the receptor proteins are processed, first by ADAM proteases, then by γ-secretase cleavage. This results in the release of a Notch intracellular domain (N-ICD), which is recruited into a nuclear transcription factor complex that activates Notch target genes. Previous *in vitro* studies showed that the Delta-like and Jagged ligand proteins can also be cleaved by the γ-secretase complex, but it remains unknown whether such processing occurs during *in vivo* vertebrate development. Here we show that mouse and human lens progenitor cells endogenously express multiple Jagged1 protein isoforms, including a Jagged1 intracellular domain. We also found that pharmacologic blockage of γ-secretase activity *in vitro* resulted in an accumulation of Jagged1 polypeptide intermediates. Finally, overexpression of an epitope-tagged Jagged1 intracellular domain displayed nuclear localization and induced the upregulation of endogenous *JAG1* mRNA expression. These findings support the idea that along with its classical role as a Notch pathway ligand, Jagged1 is regulated post-translationally, to produce multiple active protein isoforms.

## Abstract (precis)

**Summary:** The Notch pathway ligand protein Jagged1 undergoes multiple catalytic cleavages, regulated by Adam proteases and the gamma-secretase complex, during mammalian lens development, similar to Notch receptor proteins.

## INTRODUCTION

The Notch signaling pathway is highly conserved among metazoans and functions in a variety of cellular activities, including cell fate determination. One developing tissue that relies on Notch signaling is the ocular lens. The vertebrate lens consists of anterior epithelial progenitor cells that terminally differentiate into organelle-free fiber cells. This is an attractive tissue for studying complex cell–cell signaling pathways, given its simple architecture and dispensability. Vertebrate lens induction initiates at E9.0 in the mouse when the surface ectoderm thickens into a lens placode (Charlton-Perkins et al., 2011; Piatigorsky, 1981). Lens placode formation relies on signals from the underlying optic vesicle, a neuroepithelium extension of the ventral forebrain that gives rise to both the neural retina and retinal pigment epithelium (Fuhrmann, 2010). At E10.5, the lens placode invaginates into a lens pit, which subsequently detaches from the surface ectoderm as a hollow lens vesicle, comprised entirely of progenitor cells (McAvoy et al., 1999). Then posterior progenitor cells, those closest to the developing retina, elongate as they differentiate into primary lens fiber cells. At the same time, anterior progenitor cells coalesce into the anterior epithelial layer (AEL). At E14.5, the lens switches its mode of development, when AEL cells begin to migrate to the equatorial transition zone, which constitutes a boundary between progenitors and differentiated fiber cells (reviewed in Mochizuki and Masai, 2014). Upon terminal differentiation, secondary fiber cells surround the primary fiber cells that comprise the central core of the lens. Previous studies demonstrated that Notch signaling regulates progenitor cell proliferation, secondary fiber cell differentiation and helps maintain the transition zone (Azimi et al., 2018; Jia et al., 2007; Le et al., 2009; Rowan et al., 2008; Saravanamuthu et al., 2009).

After ligand binding, Notch receptors undergo proteolysis, to release the Notch intracellular domain (N-ICD) polypeptide fragment (Bray, 2006; Schroeter et al., 1998). There are two broad classes of Notch ligands, Delta or Delta-like (Dll) and Serrate/Jagged (Jag), with mammalian genomes containing multiple paralogs for each type of ligand. Ligand truncations lacking either the extracellular or intracellular domain, fail to trigger Notch receptor activation, demonstrating that the entire ligand protein is necessary for functionality (Shimizu et al., 1999; Sun and Artavanis-Tsakonas, 1996). After receptor–ligand interaction, the Notch receptor is sequentially cleaved, with each event mediated by different enzymatic activities (reviewed in Kopan and Ilagan, 2009). First, ADAM proteases remove the Notch receptor extracellular domain, followed by γ-secretase protein complex cleavage within the transmembrane region, to release the N-ICD from the plasma membrane (Struhl and Greenwald, 1999). The γ-secretase complex is comprised of four proteins: Nicastrin, Aph-1, Pen2, and Presenilin (Psen), with Psen exhibiting the catalytic activity. Moreover, the γ-secretase complex cannot cleave full length Notch receptors, likely due to steric hindrance from the receptor extracellular domain. Thus, ADAM protease activity is a prerequisite for N-ICD generation (Bolduc et al., 2016; Struhl and Adachi, 2000). Once generated, the N-ICD fragment complexes with Rbpj and Mastermind proteins, which transcriptionally activate downstream genes within the nucleus (Wilson and Kovall, 2006).

In addition to Notch receptors, the γ-secretase complex cleaves at least 90 other substrates, including the amyloid precursor protein (APP), which accumulates abnormally in Alzheimer's disease (Beel and Sanders, 2008; Haapasalo and Kovacs, 2011). Interestingly, previous *in vitro* studies showed that both Dll and Jag ligands can also be cleaved by γ-secretase (Ikeuchi and Sisodia, 2003; LaVoie and Selkoe, 2003; Six et al., 2003), and that overexpression of full length Dll or Jag in cultured cells facilitated the appearance of ligand-CTF isoforms, lacking extracellular ligand domains. This is consistent with the idea that ADAM proteases can cleave some of the Notch ligands. Furthermore, CTF isoforms accumulated after cells were treated with γ-secretase inhibitors, suggesting they are γ-secretase substrates. Although these data strongly suggest Notch pathway ligands as ADAM and/or γ-secretase substrates, the physiological relevance of ligand processing during development, homeostasis, or pathogenesis are essentially unknown. One recent *in vivo* study highlighted a role for the Jag1 intracellular domain (J1-ICD) in mouse adult cardiomyocytes (Metrich et al., 2015). Here J1-ICD overexpression reduced both Notch1 processing (N1-ICD levels), and the expression of downstream target genes *Hes1* and *Hey1/2*. Moreover, increased J1-ICD expression correlated with reduced proliferation and premature cardiomyocyte differentiation. Therefore, Notch ligand processing may modulate signaling levels, via ligand turnover from the cell membrane and/or ligand intracellular domains could have intrinsic signaling activity.

Given that Notch receptor protein processing has been extensively studied and there remains sustained interest in pharmacologic interventions to block Notch dysregulation, deeper understanding of ligand protein regulation seems warranted. Here we show the presence of Jag1 protein isoforms during *in vivo* mouse lens development and test whether ADAM protease and γ-secretase activities regulate this ligand *in vitro*, using a human lens epithelial cell line. Moreover, mouse Jag1 colocalizes with Psen1 at three key ages of lens formation, consistent with the idea that γ-secretase can also cleave this ligand. We also describe multiple Jag1 protein isoforms in other developing mouse tissues, whose sizes correspond to full length Jag1, a Jag1-CTF intermediate, and the intracellular domain, J1-ICD. We found that human lens B3 cells endogenously express and process JAG1 protein, as well as transfected, epitope-tagged Jag1 isoforms that localized to the expected subcellular compartments. We also treated lens B3 cells with DAPT, which induced the accumulation of endogenous JAG1-CTF, the predicted γ-secretase substrate. Finally, J1-ICD overexpression in B3 cells stimulated an upregulation of endogenous *JAG1* mRNA, suggesting that JAG1 protein isoforms can participate in Notch pathway feedback regulation.

## RESULTS

### Multiple Jag1 protein isoforms are present during mouse embryogenesis

**Fig. 1.**
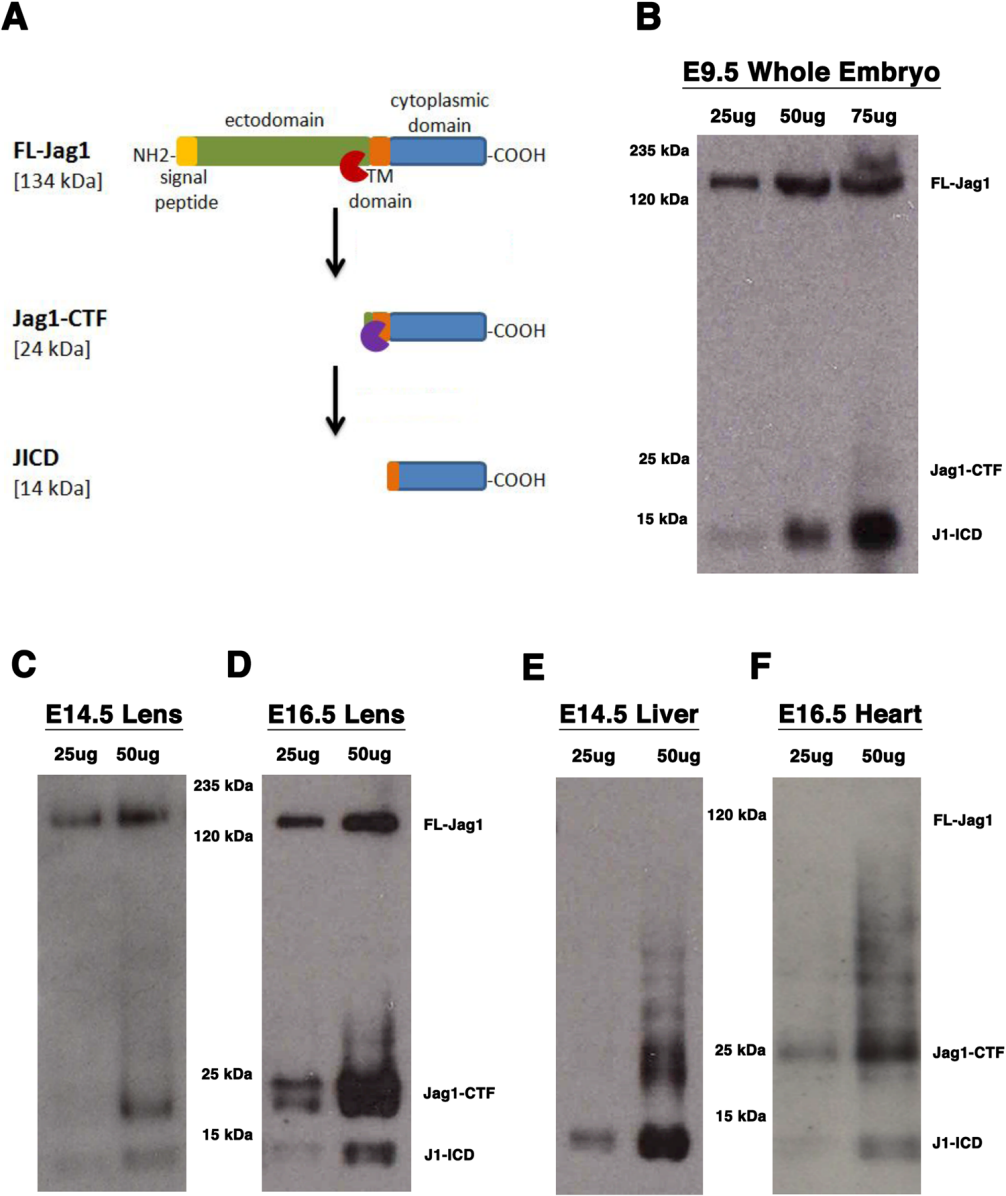
**Survey of mouse Jagged1 protein isoforms in multiple embryonic tissues.** (A) Schematic of Jag1 processed isoforms and predicted molecular weights. Red and purple symbols represent the presumed cleavages by ADAM (red) or γ-secretase (purple) activities. (B–F) Western blot analysis using a C-terminal specific Jag1 antibodies that recognizes all protein isoforms in rodent and human cells. (B) Whole E9.5 mouse embryo extract containing FL-Jag1, Jag1-CTF (seen in longer exposure), and J1-ICD, using goat anti-Jag1 antibody. All three Jag1 isoforms are also detected in the developing lens at (C) E14.5 and (D) E16.5. The only detectable isoforms in (E) E14.5 liver and (F) E16.5 heart tissues are the Jag1-CTF and J1-ICD. Panels C–F were generated using rabbit anti-Jag1 antibody. All blots are representative of three independent protein preparations and western blots (biological replicates).

Previous *in vitro* work demonstrated that epitope-tagged rat Delta-like (Dll) and Jagged1 (Jag1) proteins are cleaved by the γ-secretase complex (LaVoie and Selkoe, 2003). Given that the removal of *Jag1* activity during mouse lens development results in postnatal lens aphakia (Le et al., 2009), we hypothesized that post-translational processing of this ligand may be one mechanism for regulating its activity during embryogenesis. If the Jag1 ligand protein is processed analogously to Notch receptors, we would expect to see three distinct isoforms: full length Jag1 (FL-Jag1), Jag1 C-terminal fragment (Jag1-CTF) and Jag1 intracellular domain (J1-ICD). An ADAM-mediated juxtamembrane cleavage of FL-Jag1 removes the larger extracellular region (ectodomain) of the protein, resulting in the Jag1-CTF isoform. According to one study, ADAM17 activity is associated with Jag1 ectodomain shedding, while ADAM10 is responsible for Delta processing (LaVoie and Selkoe, 2003). After ectodomain removal, the Jag1-CTF intermediate is primed for a second cleavage within the transmembrane domain, by the γ-secretase complex, to produce the J1-ICD isoform. This Jag1 intracellular fragment, like an N-ICD, would no longer be tethered to the plasma membrane and free to translocate elsewhere inside the cell. Therefore, we used predicted cleavage site consensus sequences to estimate the molecular weights for each isoform (Fig. 1A). In mouse, FL-Jag1 is 134 kDa, the Jag1-CTF would be 24 kDa, while the J1-ICD isoform is predicted to be 14 kDa.

To determine if FL-Jag1 undergoes either type of proteolytic cleavage *in vivo*, we collected several different developing mouse tissues in which the Jag1 ligand is a key component of Notch signaling, and analyzed total protein lysates by western blotting, using a C-terminal specific Jag1 antibody (Le et al., 2009). This antibody should detect all predicted Jag1 isoforms since each retains an intact C-terminus, yet allow for identification of each proteolytic product by its distinct molecular weight. We probed E9.5 whole mouse embryo extracts, a developmental age when there is very high Notch activity throughout the body. We detected both FL-Jag1 and a fragment matching the predicted size of the J1-ICD (Fig. 1B). Longer exposure times did reveal the Jag1-CTF band in whole embryo total protein extracts (data not shown). By contrast, in both the E14.5 and E16.5 lens, all three predicted Jag1 isoforms were readily visualized (Fig. 1C,D). Conversely, in other tissues, only the lower molecular weight Jag1 protein isoforms were detected. For instance, the Jag1-CTF and J1-ICD were abundant in E14.5 liver and E16.5 heart tissue lysates (Fig. 1E,F). We also consistently noted a protein doublet for the Jag1-CTF, suggesting the possibility of two potential cleavage start sites, or that this protein undergoes additional post-translational modifications. Intriguingly, each band in the doublet displayed different signal strengths, depending on the tissue analyzed. We also performed another western blot with all embryonic tissue lysates present on single blot and then reprobed it with a β-actin antibody, for direct comparison of protein loading (Fig. S1).

Because the Jag1-CTF was consistently present in embryonic lens extracts, we wished to understand if production of this isoform depends on the activity of particular Adam proteins. Although there are 40 different *Adam* genes in mammalian genomes, two family members have consistently been associated with the Notch signaling pathway, *Adam10* and *Adam17* (Groot and Vooijs, 2012). Interestingly, the *Drosophila* Adam10 homolog, Kuzbanian, cleaves the Delta ligand in fly embryos (Qi et al., 1999). Therefore, we compared Jag1 protein isoforms in the absence of *Adam10* or *Adam17*, by western blotting of total protein extracts from E14.5 mouse lenses collected from control and mutants (Fig. S2). Conditionally mutant lens tissues were generated using the same Cre-lox strategy employed in previous studies of Notch signaling in the developing mammalian lens (Azimi et al., 2018; Le et al., 2009; Rowan et al., 2008), by using Le-Cre to delete either *Adam10* or *Adam17*, producing heterozygotes and homozygotes in normal Mendelian ratios. We noted comparable levels of Jag1-CTF in E14.5 control, Le-Cre;*Adam17^CKO/+^* and Le-Cre;*Adam17^CKO/CKO^* lanes (Fig. S2A), suggesting that Adam17 does not cleave Jag1. However, lens extracts from E14.5 Le-Cre;*Adam10C^KO/CKO^* mutants completely lacked Jag1-CTF protein (Fig. S2B). By comparison, Notch2 receptor protein processing was also affected in Le-Cre;*Adam10C^KO/CKO^* mutants, where there was reduced levels of the Notch2 extracellular truncation fragment (N2-EXT), which is normally generated by Adam cleavage (Fig. S2C). These data suggest that Adam10 cleaves both Notch2 and Jag1 proteins in the embryonic mouse lens.

### Jag1 and Psen1 protein co-expression during lens development

In mammals, there are two *Psen* genes, *Psen1* and *Psen2*, that are both present in the developing mouse lens (Azimi et al., 2018), although each γ-secretase protein complex only contains one Psen protein paralog (De Strooper et al., 2012). In many tissues there are different requirements for Psen1- versus Psen2-containing γ-secretase complexes, best illustrated by the early lethality of *Psen1* germline mutants, whereas *Psen2* mutants are adult viable (Donoviel et al., 1999; Shen et al., 1997). Although both Psen proteins catalyze proteolysis, Psen1 and Psen2 display different substrate specificities that are linked to distinct intracellular localizations (Sannerud et al., 2016). The Psen1 protein is more highly expressed at the plasma membrane, whereas Psen2 is largely found in the membranes of late endosomes and lysosomes. Thus, it is not surprising that Psen1-containing γ-secretase complexes are more efficient at cleaving the plasma membrane protein Cdh2/N-Cadherin than those complexes containing the Psen2 protein (Sannerud et al., 2016).

**Fig. 2.**
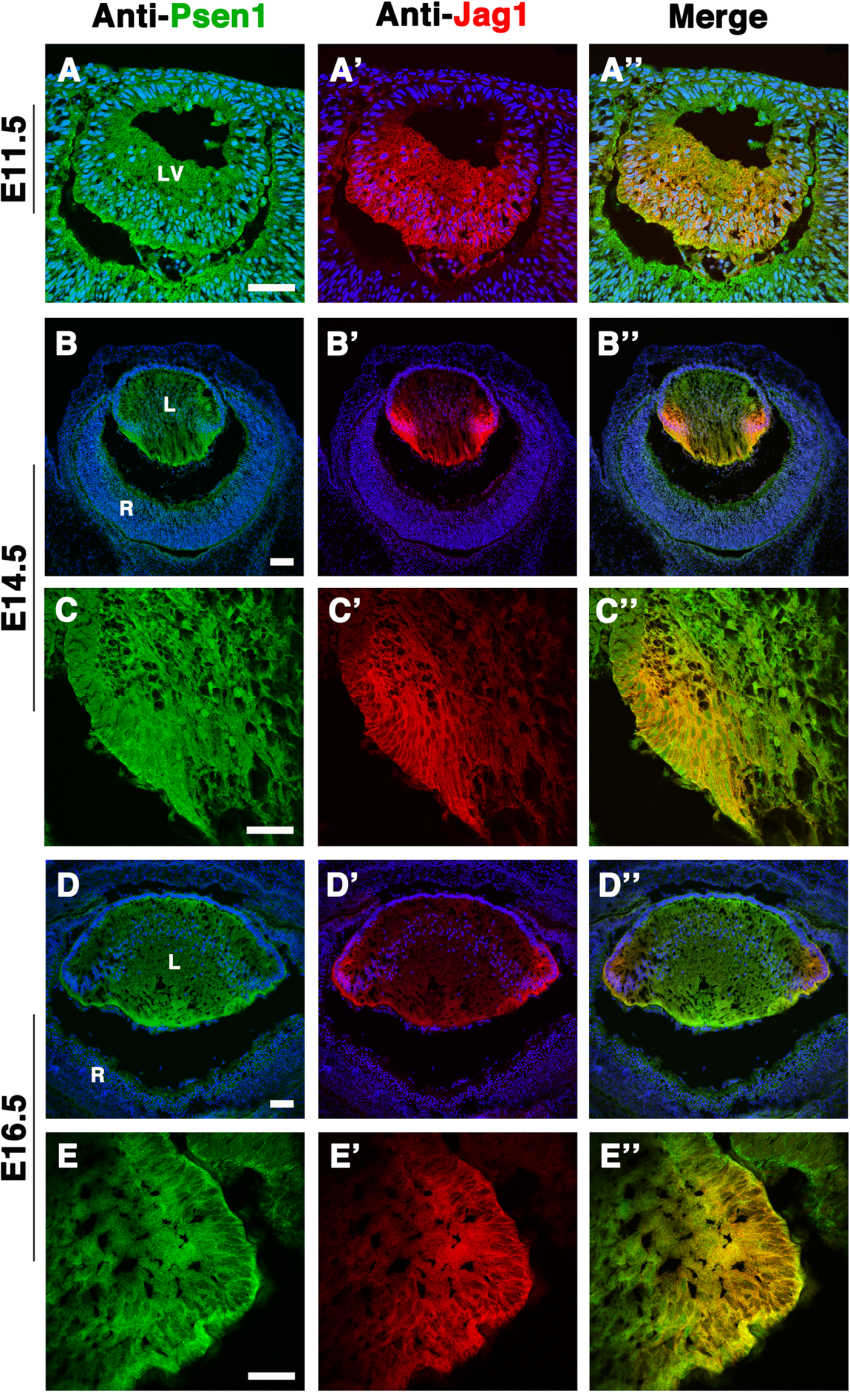
**Jag1 and Psen1 co-localization in the prenatal mouse lens.** Double antibody labelling of wild-type E11.5 lens cryosections (A–A″) demonstrating that Psen1, being ubiquitously expressed in the developing lens (Azimi et al., 2018), has overlap with the Jag1 protein domain (visualized using goat anti-Jag1) that marks the posterior half of the lens vesicle, where cells are beginning to differentiate into primary fiber cells. At E14.5 (B–B″) and E16.5 (D–D″) Jag1 and Psen1 colocalization becomes confined to the Jag1-expressing domain that is now restricted at the lens transition zone, seen more clearly in close-up images (C–C″) and (E–E″), respectively. *n*=4 biological replicates at E11.5 and E14.5; *n*=2 biological replicates at E16.5. Anterior is up in all panels. LV, lens vesicle; L, lens; R, retina. Scale bar: in A,C,E = 50 µm, in B,D = 100 µm.

Since the Jag1 protein has transmembrane domains and localizes to the cell membrane (Grochowski et al., 2016), we wished to compare its expression pattern to that of Psen1, using previously validated specific antibodies (Azimi et al., 2018). If Psen1-containing γ-secretase complexes cleave the Jag1-CTF to produce a J1-ICD fragment, then Jag1 and Psen1 should colocalize. We performed double antibody labeling at different lens developmental ages, ranging from E11.5 to E16.5. At E11.5, Psen1 and Jag1 expression overlapped in posterior lens progenitor cells as they initiate primary fibergenesis (Fig. 2A–A″). However, once secondary fibergenesis begins at E14.5 and onwards, Psen1 and Jag1 were both observed in the transition zone within nascent, secondary fiber cells (Fig. 2C–C″,E–E″). Thus, Psen1 and Jag1 coexpression further supports our idea that during *in vivo* lens formation Jag1 appears to be a γ-secretase substrate.

### Endogenous JAG1 protein processing in a human lens epithelial cell line

**Fig. 3.**
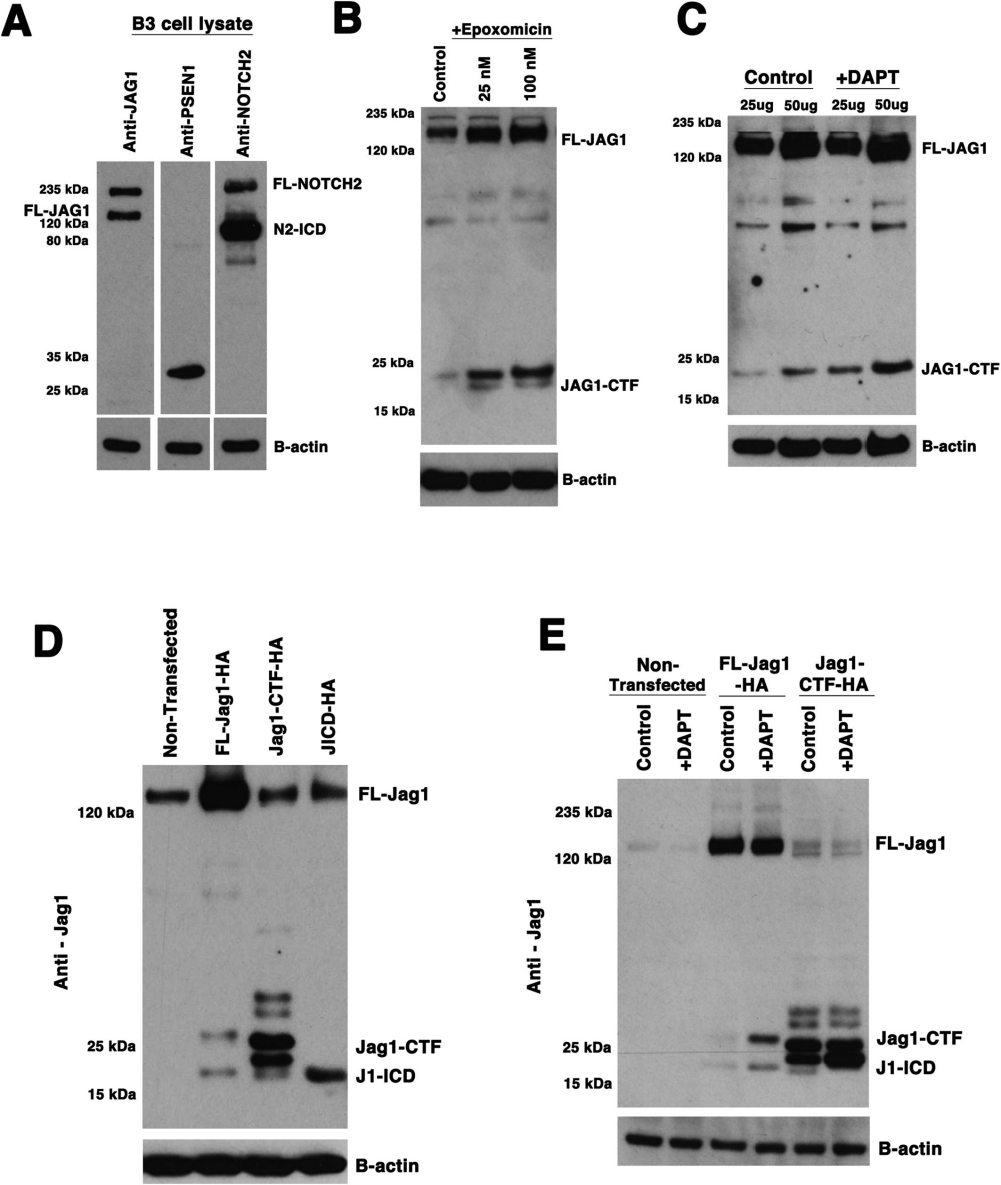
**DAPT-treatment of human lens B3 cells induces changes in endogenous JAG1 protein processing.** (A) Endogenous JAG1, NOTCH2 and PSEN1 expression in B3 cell lysates. Each lane/strip blot contains 50 µg of B3 cell total protein that was separately probed with rabbit anti-Jag1, rabbit anti-Psen1 (N-terminus), or rat anti-Notch2 to reveal expression of FL-JAG1, PSEN1 and NOTCH2 proteins. The JAG1-CTF isoform was observable in longer exposures (not shown, see control lanes in Fig. 3B,C). The NOTCH2 full length and N2-ICD isoforms were clearly detectable. (B–C) B3 cells treated with different concentrations of Epoxomicin versus DMSO (B), or 10 µM DAPT versus DMSO (C) for 24 h and cell lysates analyzed for JAG1 protein isoforms via western blot using rabbit anti-Jag1 antibody. (D) Western blotting of B3 cells transfected with C-terminal HA-tagged isoforms of rat Jag1 protein (50 µg total protein loaded per lane; blot probed with rabbit anti-Jag1 antibody). (E) B3 cells transfected with either FL-Jag1-HA of Jag1-CTF-HA constructs, followed by treatment with 10 µM DAPT (versus DMSO alone) for 24 h. Western blot probed with rabbit anti-Jag1. In all panels, blots were reprobed with mouse anti β-Actin as a loading control. Each blot is representative of *n*=3 independent biological replicates.

Given the relatively low level of J1-ICD protein detectable in protein extracts from 12 pooled mouse E14.5 lenses, we took advantage of a well-characterized human lens epithelial cell line (HLE-B3, termed B3 here) (Andley et al., 1994) to analyze JAG1 protein post-translational processing more deeply. These cells endogenously express the relevant Notch signaling pathway components (Fig. 3A). We also detected endogenously processed NOTCH2 receptor protein (N2), with the N2-ICD isoform being abundant in B3 cell protein extracts. By comparison, human B3 cells endogenously express the J1-ICD isoform at relatively low levels, similar to mouse embryonic lenses (Fig. 3A–C, and data not shown). The lower levels of J1-ICD in both B3 cells and prenatal mouse lenses might be attributed to a short half-life for this isoform, and/or it may undergo further processing, such that it escapes detection with a C-terminal antibody. To test the first idea, B3 cells were treated with epoxomicin, a potent proteasome inhibitor (Meng et al., 1999) for 24 h, across a fourfold concentration range and the cell lysates assayed by western blot for the presence of different JAG1 isoforms (Fig. 3B). Although the epoxomicin treatment did not enhance our visualization of the J1-ICD, we did see an increase of JAG1-CTF expression in treated cells. This implies that in the mammalian lens, the Jag1-CTF isoform may normally be turned over via proteasomal degradation.

Next, we wished to test for γ-secretase complex activity in B3 cells by treating them with N-[N-(3,5-difluorophenacetyl)-L-alanyl]-S-phenylglycine t-butyl ester (DAPT), which binds to the C-terminus of Psen proteins and effectively blocks Notch receptor intracellular domain production (Dovey et al., 2001; Geling et al., 2002; Morohashi et al., 2006). Thus, by analogy, administration of the γ-secretase inhibitor DAPT in B3 cells should block J1-ICD production. However, since consistent visualization of the endogenous J1-ICD isoform is challenging, we instead asked whether DAPT-treated B3 cells accumulate more JAG1-CTF, the presumed substrate of the γ-secretase complex (Fig. 3C). We noted an accumulation of the JAG1-CTF, similar to previous *in vitro* assessments of JAG1 protein processing (LaVoie and Selkoe, 2003; Metrich et al., 2015). To independently demonstrate the ability of B3 cells to produce JAG1 protein isoforms, we transfected B3 cells with three different constructs containing HA epitope-tagged forms of rat FL-Jag1, Jag1-CTF, and J1-ICD (LaVoie and Selkoe, 2003), which each electrophorese at slightly higher molecular weights than their endogenous counterparts. We used both specific Jag1 antibodies and an anti-HA antibody to detect the tagged isoforms (Fig. 3D; Fig. S3). Interestingly, cell lysates from both FL-Jag1-HA and Jag1-CTF-HA transfection contained the J1-ICD isoform (Fig. 3D, compare with J1-ICD-HA transfected lane). Paradoxically, the anti-HA antibody probed western blot also detected the epitope-tagged J1-ICD in lysates from in Jag1-CTF-HA transfection, but, not in the FL-Jag1-HA transfected lysates (Fig. S3). This might suggest that the J1-ICD band we observe with the anti-Jag1 antibody upon transfection with FL-Jag1-HA (Fig. 3D) stems from endogenous protein in the cell being preferentially processed into J1-ICD. It could be that the HA-tag on the FL-Jag1-HA protein sterically hinders the γ-secretase complex from binding and/or cleaving this isoform, thereby allowing it to only act on the endogenous Jag1 protein inside the cell to generate untagged J1-ICD. Alternatively the HA-tag may be selectively cleaved off of only the FL-Jag1-HA by an unknown mechanism.

We then used DAPT to block γ-secretase activity and ask to what extent this affected the relative expression of the different tagged Jag1 isoforms, via western blotting of cell protein lysates. We found that DAPT-mediated inhibition of γ-secretase in B3 cells transfected with the FL-Jag1-HA resulted in an accumulation of both Jag1-CTF-HA and J1-ICD-HA isoforms (Fig. 3E). Because Jag1-CTF is the presumed substrate of γ-secretase activity, accumulation of this tagged isoform after DAPT treatment (Fig. 3E) is consistent with an increase in endogenous JAG1-CTF in non-transfected cells (Fig. 3C). However, we anticipated that J1-ICD expression would be missing. Nonetheless, we observed that DAPT treatment following Jag1-CTF-HA transfection resulted in more JAG1-CTF and a loss of the J1-ICD band (Fig. 3E).

### Subcellular localization of epitope-tagged Jag1 proteins isoforms in B3 cells

**Fig. 4.**
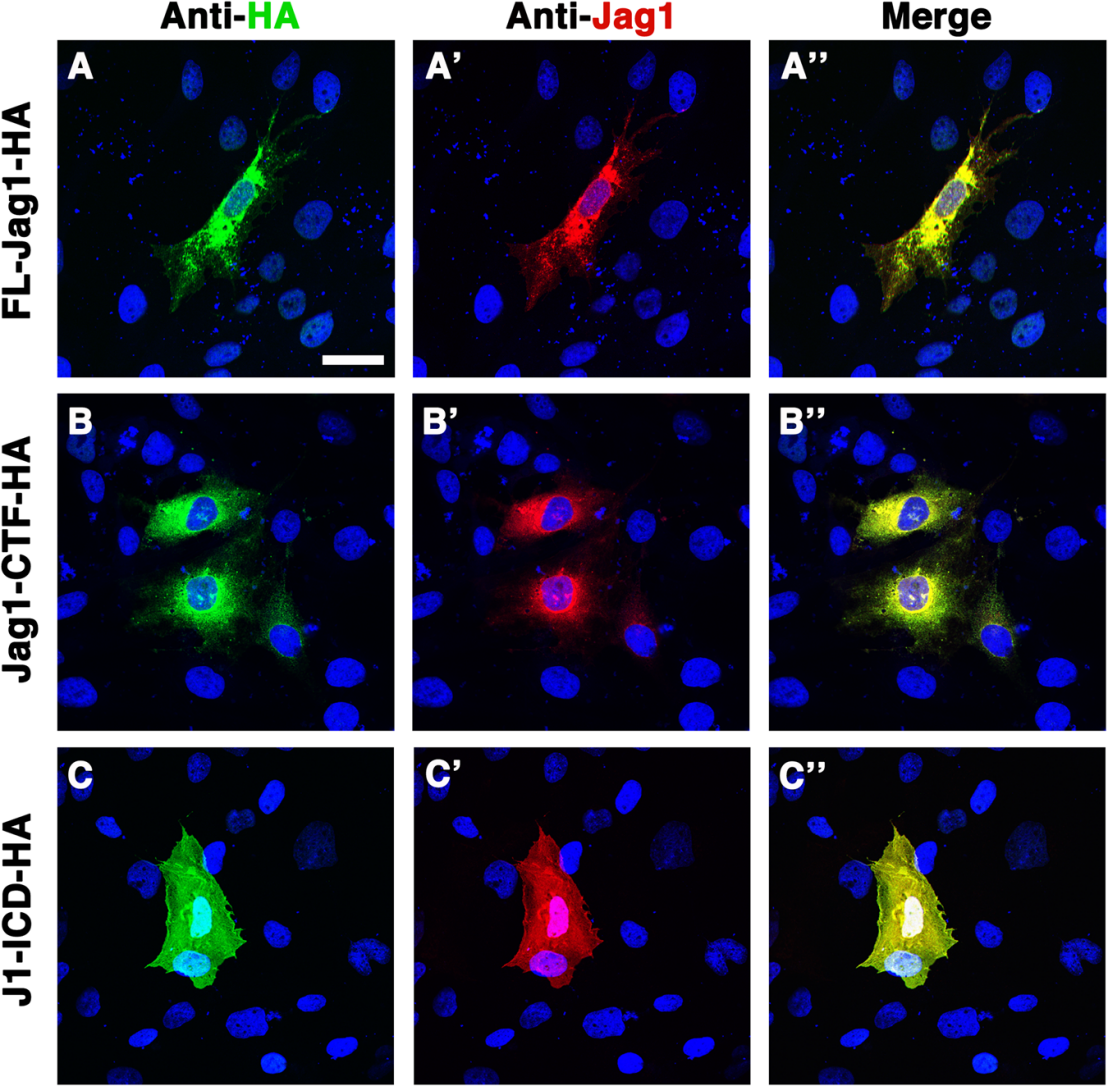
**Different subcellular localizations of transfected epitope-tagged Jag1 constructs.** In transfected B3 lens progenitor cells co-labeled with rabbit anti-HA (green) and goat anti-Jag1 (red) antibodies, FL-Jag1-HA is present throughout the cell but excluded from nucleus (A–A″). The distribution of Jag1-CTF-HA is similar to the full length tagged protein except also uniquely displays some nuclear localization (B–B″). J1-ICD-HA transfected cells exhibit strong nuclear expression (C–C″). *n*=3 biological replicate experiments for each construct. Scale bar: 50 µm.

Intramembrane cleavage by γ-secretase releases the N-ICD fragment from the plasma membrane, facilitating its accumulation in a nuclear protein complex that regulates the transcription of target genes. Using B3 cells transfected with epitope-tagged Jag1 constructs, we compared the subcellular localization of the FL, CTF and ICD Jag1 isoforms. Similar to the Notch receptor N-ICD fragment, the J1-ICD polypeptide contains a nuclear localization signal that is conserved from *Drosophila* Serrate to human JAG1 and JAG2 proteins. Using specific antibodies for either the HA-epitope tag (green) or the Jag1 C-terminus (red), we co-immunolabeled the transfected B3 cells. As a control, non-transfected B3 cells did not label with the anti-HA-antibody, while the anti-Jag1 antibody faintly labeled the plasma membrane (data not shown). We also confirmed that there was no cross-reactivity among the secondary antibody reagents by omitting the relevant primary reagent in replicate experiments (not shown). Cells transfected with the FL-Jag1-HA construct showed labeling by both antibodies throughout the cell cytoplasm and at the cell surface, (Fig. 4A–A″). Those cells transfected with Jag1-CTF-HA displayed a similar labeling pattern to those with FL-Jag-HA, but with some unique expression within the DAPI-labeled (blue) nuclei (Fig. 4B–B″). Interestingly J1-ICD-HA transfected cell nuclei were more obviously labeled by both antibodies (Fig. 4C–C″). Based on our observation that Jag1-CTF-HA transfection appears to enhance the accumulation of exogenous J1-ICD (Fig. 3D), it is possible that the additional nuclear expression in Jag1-CTF-HA transfected cells is attributable to enhanced production of J1-ICD (both endogenous and epitope-tagged) in these cells. Overall, we conclude that the J1-ICD isoform is capable of localizing in B3 nuclei.

### J1-ICD-HA protein overexpression induces endogenous *JAG1* transcription

**Fig. 5.**
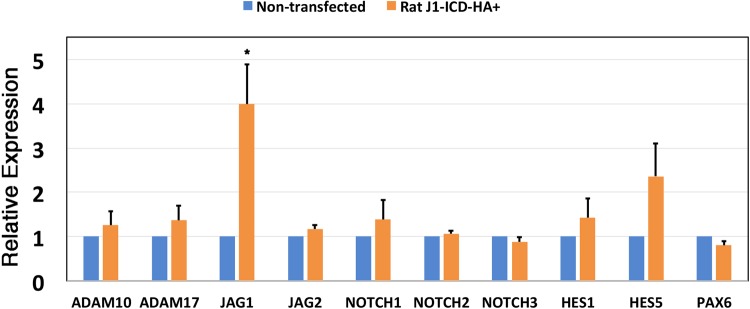
**Comparison of mRNA expression levels after JICD-HA transfection of B3 cells.** The relative mRNA levels of various Notch pathway genes, and the PAX6 transcription factor, between B3 control versus J1-ICD transfected cells. The relative expression level of each mRNA was determined by qPCR, normalized to β-actin, and a non-transfected control. The graphed expression values are the mean of three independent biological replicate experiments, each performed in technical triplicate, with the error bar representing the s.e.m. *P≤0.05. Statistical significance was determined by one-way ANOVA for the entire dataset, as well as by comparing control and transfected data using a two-tailed Student's *t*-test using Microsoft Excel 2016 Analysis ToolPak.

The presence of nuclear J1-ICD protein raises the possibility that it might induce a cellular response, and so represent an alternative mechanism for feedback regulation within the signaling pathway. Expression of ligand intracellular domain isoforms was previously implicated in the downregulation of Notch target genes such as *Hes1*, *Hey1*, and *Hey2* (Metrich et al., 2015); the activation of the AP-1 element (LaVoie and Selkoe, 2003); the cellular transformation of RKE cells (Ascano et al., 2003); an enhancement of Smad-dependent transcription (Hiratochi et al., 2007); and an increase *Cdh1/E-Cadherin* gene expression (Delury et al., 2013). Here we wished to test the impact of J1-ICD overexpression on the expression levels of other Notch pathway genes. We used qRT-PCR to quantify mRNA expression levels between non-transfected versus J1-ICD-HA-transfected B3 cells. We also monitored the expression of the ocular factor, *PAX6* (Fig. 5). Among the ten genes tested, only *JAG1* mRNA levels were significantly affected, showing a fourfold increase. To ensure that only human B3 cell *JAG1* expression was assayed, we used PCR primers that amplify 5′ sequences not present in the J1-ICD isoform. Moreover, the J1-ICD-HA construct utilizes rat *Jag1* gene sequences. Overall, our data suggest that Jag1 protein can self-regulate its own expression, via the activity of a functional J1-ICD isoform.

## DISCUSSION

The objective of this study was to test the hypothesis that the Jag1 ligand protein is cleaved analogously to the Notch receptor during mammalian lens development, and to explore possible functions of a J1-ICD polypeptide. Here we demonstrate the expression of distinct Jag1 protein isoforms, in multiple developing mouse tissues, including the forming ocular lens. Similar to Notch receptors, FL-Jag1 undergoes ectodomain shedding, via ADAM protease activity, to generate a Jag1-CTF intermediate, as well as γ-secretase complex-mediated intramembrane proteolysis to produce a J1-ICD polypeptide. We also found that the latter protein species can be localized to the cell nucleus where it feeds back onto its own gene transcription.

Previous *in vitro* studies have also assessed whether ADAM proteases can cleave Jag1. One study pointed to ADAM17 as responsible for Jag1-CTF production based on the finding that an ADAM17-specific inhibitor resulted in lower levels of Jag1-CTF while an ADAM10-specific inhibitor did not alter Jag1-CTF levels (LaVoie and Selkoe, 2003). However, another study used siRNA knockdown to show that ADAM10 is more effective at cleaving FL-Jag1 than ADAM17 (He et al., 2014). Our *in vivo* lens mutant tests, albeit preliminary for *ADAM10* mutants, show that in the developing mouse lens, Jag1-CTF generation is dependent on ADAM10 and not ADAM17. This is at odds with LaVoie and Selkoe, who used pharmacologic inhibitors in cultured cells, which may not have had sufficient specificity for a particular ADAM protease. Alternatively, the different outcomes could be explained by cell-type specific activities of individual ADAM proteins.

Our finding that DAPT treatment fails to inhibit J1-ICD production in B3 cells transfected with FL-Jag1-HA, but not Jag1-CTF-HA, was puzzling (Fig. 3E). If γ-secretase solely cleaves Jag1-CTF to produce a J1-ICD, blocking this activity via DAPT inhibition should prevent the accumulation of the J1-ICD protein. One possibility may be that Jag1 is so highly over-expressed in the cell that either the concentration of DAPT or treatment period were insufficient to effectively block substrate from being processed in the cell, resulting in some J1-ICD in our extracts. Treating cells with DAPT either prior or concurrently with transfection might circumvent Jag1 processing prior to DAPT blockage of γ-secretase activity. Alternatively, γ-secretase may only act on a specific Jag1-CTF protein product. Jag1-CTF likely undergoes other post-translational modifications (or has two possible cleavage start sites), since it is detected as a protein doublet by western blot. By referring to the higher molecular weight band as Jag1-CTF-A and the lower molecular weight band as Jag1-CTF-B, we note that overexpression of FL-Jag1-HA led to the production of only Jag1-CTF-A, whereas transfection of the Jag1-CTF-HA construct produced both Jag1-CTF-A and Jag1-CTF-B, like the *in vivo* doublet (Fig. 3D). Moreover, DAPT treatment of the Jag1-CTF-HA transfected cells resulted in an accumulation of only the Jag1-CTF-B band (Fig. 3E). It would be interesting in the future to search for other types of Jag1 protein post-translational modifications, one of which might produce a preferential substrate for the γ-secretase complex.

Ligand proteolysis affects the amount of FL-ligand present at the cell surface. For this reason, this process has been postulated to modulate Notch signaling levels, by controlling ligand availability for Notch receptor binding. Our data shows that ligand processing may also stimulate transcription of ligand mRNA. This finding is at odds with another study that showed decreased *Jag1* mRNA expression after J1-ICD overexpression in the mouse neonatal heart (Metrich et al., 2015). One critical difference is that the cardiac J1-ICD isoform is a 20 kDa fragment, whereas we detected a 14 kDa isoform in the lens. Interestingly, based on protein domains and amino acid sequence of Jag1, a 20 kDa Jag1 proteolytic fragment is consistent with an isoform that retains a fully intact transmembrane domain. Thus, J1-ICD overexpression in cardiac myocytes might produce a Jag1-CTF that is still membrane-tethered, unlike the isoforms produced in the lens. It is also possible that J1-ICD promotes distinct cellular responses in different tissues or during development (this study) versus in adult tissues (Metrich et al., 2015). Finally, our finding that overexpressed J1-ICD can induce endogenous *JAG1* mRNA expression might contribute to ligand recycling since processed proteins are potentially turned over more rapidly yet simultaneously promote the production of new ligand.

In the lens, *Jag1* activity maintains both AEL progenitor cell proliferation and secondary fiber cell differentiation in the equatorial transition zone (Le et al., 2009). Moreover, Le-Cre;*Jag1^CKO/CKO^* mice exhibit more severe adult lens phenotypes than those of other Notch pathway mutant mice, for example Le-Cre;*Rbpj^CKO/CKO^* and Le-Cre;*Notch1^CKO/CKO^*;*Notch2^CKO/CKO^* mutants (Azimi et al., 2018; Le et al., 2009; Rowan et al., 2008). Data presented here are consistent with Jag1 protein regulation post-translationally via proteolytic processing. This suggests a possible mechanism by which Jag1 could also act in a Notch-independent manner to regulate other aspects of lens formation. Interestingly, a previous study correlated J1-ICD transfection in HEK cells with an increase in *Cdh1* transcription (Delury et al., 2013). Because *Jag1* mutant embryonic lenses displayed a severe loss of Cdh1+ AEL cells (Le et al., 2009), future studies should look more deeply for a potential relationship between Jag1 protein processing and Cdh1 expression.

It still unknown to what extent Notch ligand ICD polypeptides can influence gene transcription (directly or indirectly), since the endogenous levels of this isoform appear to be quite low, at least during mouse embryogenesis. However, ligand ICD amino acid sequences contain a PDZ recognition motif that might facilitate protein–protein interactions (Forghany et al., 2018; Hock et al., 1998); and this PDZ domain is required during Jag1-mediated transformation of rat kidney epithelial (RKE) cells, suggesting ligand ICDs as possessing inherent functionality (Ascano et al., 2003). It would be informative to search for other proteins that can physically interact with J1-ICD and perform RNA-seq to quantify gene expression level changes after J1-ICD overexpression. Deeper understanding of the potential roles of Jag1 protein isoforms have a good likelihood of informing mechanisms of bi-directional Notch signaling.

## MATERIALS AND METHODS

### Animals

Wild-type CD-1 mice were obtained from Charles Rivers. Le-Cre Tg/+ mice were maintained on an FVB/N background and PCR genotyped as described (Ashery-Padan et al., 2000). *Adam17^tm1.2Bbl^* mice (*Adam17^CKO/CKO^*) were obtained from the Jackson Laboratory, maintained on a mixed 129-C57BL/6 background and genotyped using JAX online genotyping protocols. Embryonic mouse lens tissues from Le-Cre;*Adam10C^KO+/^*×*Adam10^CIO/CKO^* timed matings were provided and genotyped by Duska Sidjanin (Medical College of Wisconsin). The embryonic age was determined by vaginal plug detection at day E0.5.

### Ethics statement

All mice were housed and cared for in accordance with guidelines provided by the National Institutes of Health, Bethesda, Maryland, and the Association for Research in Vision and Ophthalmology, and conducted with approval and oversight from the UC Davis Institutional Animal Care and Use Committees.

### Plasmids and transfections

Rat FL-Jag1-HA (pBOS-SN3T), Jag1-CTF-HA (pEF6/V5-His) and JICD-HA (pEF6/V5-His) HA-tagged expression plasmids were provided by Matthew LaVoie (LaVoie and Selkoe, 2003) and verified using Sanger DNA sequencing. Each plasmid was transfected into B3 cells using the Fugene6 (Promega, Cat#:E2691) protocol at a 3:1 volume ratio of Fugene6 transfection reagent relative to that of the DNA. For immunocytochemistry experiments, 2.0×10^5^ to 2.5×10^5^ B3 cells were plated per well of a 2-well chamber slide (Thermo Fisher Scientific, Cat#:177429) and transfected with 40 ng of plasmid after 24 h (∼60–70% confluency). In experiments generating material for western blot analysis, 1.0×10^6^ to 1.5×10^6^ B3 cells were plated onto a 100 mm tissue culture plate and transfected with 5 µg of plasmid after 24 h (60–70% confluency). Cells remained in transfection mix media for 48 h at 37°C and 5% CO_2_ before either fixing for staining protocol or harvesting for protein extraction via cell-scraping in cold PBS plus cOmplete mini protease inhibitor tablet (Sigma-Aldrich, Cat#:11836153001), spinning briefly, and snap-freezing the cell pellet.

### Cell culture and treatment

The immortalized human epithelial lens cells HLE-B3, termed B3 here, [American Type Culture Collection (ATCC Cat#:CRL-11421)], originally developed by Andley et al. (1994), were previously purchased by Tom Glaser and provided to us at passage 4. Cells were cultured in Dulbecco's Modified Eagle Medium (DMEM) containing 4.5 g/l D-Glucose, L-Glutamine, and 110 mg/l Sodium Pyruvate (Life Technologies, Cat#:11995-065), supplemented with 10% FBS (Atlanta Biologicals, Cat#:S11150) and penicillin (100 units/ml), and grown in a humidified incubator supplied with 5% CO_2_ at 37°C. Cells tested negative for mycoplasma and Fig. 5 qPCR data authenticate their human origin. For pharmacological treatment assays, 1.0×10^6^ to 1.5×10^6^ B3 cells were plated onto a 100 mm tissue culture plate and transfected as described above. At 24 h post-transfection, cells were treated with the γ-secretase inhibitor DAPT (APExBIO, Cat#:A8200) at a concentration of 10 µM in DMSO, or DMSO alone in controls, for an additional 24 h before being harvested as stated above. For epoxomicin (APExBIO, Cat#:A2606) treatments, cells were plated on 100 mm plates in medium supplemented with different concentrations of epoxomicin or DMSO control for 24 h before being harvested.

### Immunohistochemistry/immunocytochemistry

Wild-type embryonic tissue was fixed in 4% paraformaldehyde/PBS for 1 h on ice, processed by stepwise sucrose/PBS incubation ranging from 5–15%, and embedded in OCT, then 10 μm frozen sections were generated for marker analyses as described in Brown et al. (1998). Transfected cells grown on chamber slide coverslips were fixed with 4% paraformaldehyde/PBS for 10 min and permeabilized with 0.5% Triton X-100/PBS for 5 min at room temperature. Following PBS washing, cells were blocked in 4% milk/TST for 1 h. As with tissue sections, cells cultured in slide chambers were antibody labeled as described in Brown et al. (1998). The primary antibodies used were rabbit anti-HA (1:200, Santa Cruz Biotechnology, Cat#:sc-805-discontinued; AB 631618), goat anti-Jag1 (1:250, Santa Cruz Biotechnology, Cat#:sc-6011-discontinued; AB 649689), and rabbit anti-Psen1 (1:100, Santa Cruz Biotechnology, Cat#:sc-7860-discontinued; AB 2170581). Slides were subsequently incubated with directly conjugated AlexaFluor secondary antibodies (1:400, Jackson ImmunoResearch or Thermo Fisher Scientific) or biotinylated secondary antibodies (1:500, Jackson ImmunoResearch or Thermo Fisher Scientific) followed by AlexaFluor conjugated streptavidin (1:500, Jackson ImmunoResearch). Nuclear staining was performed with DAPI (1:1000 dilution of a 1 mg/ml solution, Sigma-Aldrich, Cat#:28718-90-3).

Antibody labeled cryosections and chamber slide mounts were imaged using a Leica DM5500 microscope, equipped with a SPEII solid state confocal and processed using Leica LASAF and Adobe Photoshop (CS4) software programs. All images were equivalently adjusted for brightness, contrast and pseudo-coloring. For embryonic sections, three individuals were analyzed, using at least two sections per individual. For subcellular localization analyses in cultured cells, three independent transfection experiments per isoform were analyzed.

### Western blotting

E14.5 or E16.5 CD-1 mouse littermate lenses were hand dissected in cold PBS, pooled together, and snap frozen. E14.5 individual pairs of mouse lenses in the *ADAM17* allelic series were hand dissected away from other ocular tissues, harvested in cold PBS, snap frozen. Those of identical genotypes were subsequently pooled just prior to lysis step. Upon thawing, the lenses were lysed in RIPA buffer (150 mM NaCl, 50 mM Tris pH8, 1% NP40, 0.5% DOC, 0.1% SDS) plus cOmplete mini protease inhibitor tablet (Sigma-Aldrich, Cat#:11836153001) for 2 h with micro stir bar agitation. B3 cell pellets were thawed on ice in RIPA buffer, followed by sonication with a micro-tip sonicator. All lysates were spun down at 15,800 RCF, quantified by Bradford assay (Bio-Rad Protein Assay, Cat#:500-0006), and 25 µg (or higher amounts as indicated on particular blots) of total protein were loaded onto NuPage 4-12% Bis-Tris gel (Invitrogen, Cat#:NP0322BOX), electrophoresed in MES running buffer (Invitrogen, Cat#:NP0002-02), and transferred onto 0.2 µm nitrocellulose membranes (Invitrogen, Cat#:LC2000). Blots were blocked in 5% milk/0.1 M Tris (pH 7.4)/0.15 M NaCl/0.1% Tween20. Protein detection was performed with subsequent primary antibodies: mouse anti-β-actin (1:3000, Sigma-Aldrich, Cat#:A1978; AB 476692), rat anti-HA (1:2000, Sigma-Aldrich, Cat#:11867423001;AB 390918), goat anti-Jag1 (1:2000, Santa Cruz Biotechnology, Cat#:sc-6011-discontinued; AB 649689), and rabbit anti-Jag1 (1:1000, Santa Cruz Biotechnology, Cat#:sc-8303-discontinued; AB 649685). Blots were incubated with HRP-conjugated secondary antibodies from Jackson ImmunoResearch. ECL kit (Thermo Fisher Scientific, Cat#:34078) was used for visualization as described by manufacturer. Signals were detected using the Konica Minolta SRX-101A medical film processor.

### RNA purification and quantitative PCR analysis

Total B3 cell RNA was isolated using the Zymo Research Quick RNA miniprep kit (Cat#:R1055). RNA concentrations were measured with a Qubit 3.0 Fluorometer and Molecular Probes Qubit RNA HS Assay kit (Cat#:Q32852). 100 ng of total RNA was reverse transcribed into cDNA via the Bio-Rad iScript cDNA Synthesis kit (Cat#:170-8891) and used for qPCR analysis using Applied BioSystems Fast Sybr Green Master Mix (Cat#:4385614) and primer sets listed in Table S1 on an Applied Biosystems StepOnePlus machine. Relative Quantification (RQ) values were calculated using comparative CT method (Livak and Schmittgen, 2001) with β-Actin as a normalization control. Statistical significance was determined by one-way ANOVA or a two-tailed Student's *t*-test using Microsoft Excel 2016 Analysis ToolPak, with *P*-values <0.05 significant for the datasets in which *n*=3 biological replicates.

## Supplementary Material

Supplementary information
