# Supplementary material for: Jagged1 protein processing in the developing mammalian lens
Source: Biol Open. 2019 Mar 19;8(3):bio041095. doi: 10.1242/bio.041095 (PMC6451331; doi:10.1242/bio.041095)
Supplement: Supplementary information [file biolopen-8-041095-s1.pdf]

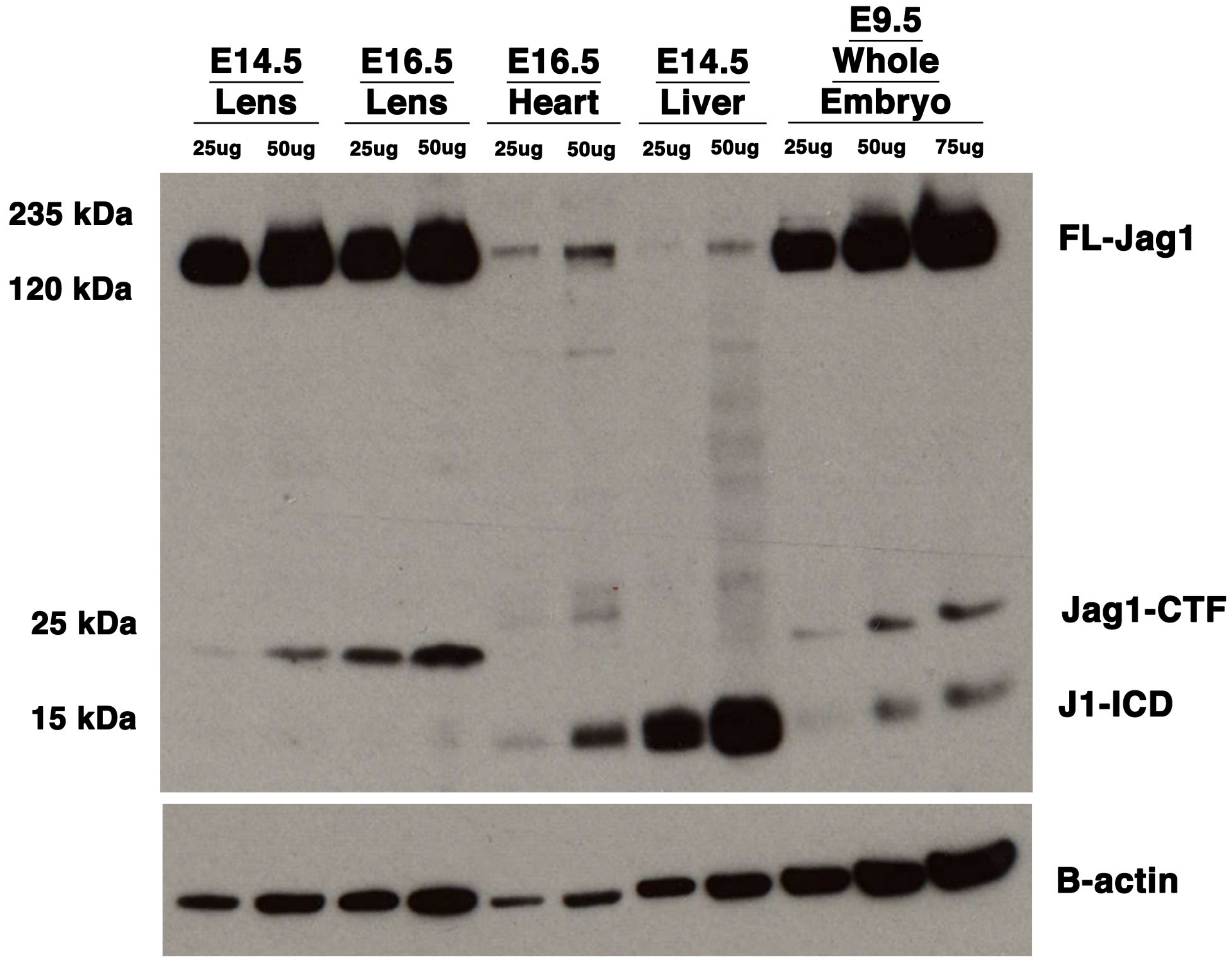

Figure S1. Loading controls for mouse embryonic tissues analyzed in Figure 1. Western blot analysis using a C-terminal specific Jag1 antibody that recognizes all protein isoforms. Samples assessed include wild type E14.5 and E16.5 lenses, E14.5 liver, E16.5 heart and E9.5 whole mouse embryos (probed with rabbit anti-Jag1). Blot reprobed with mouse anti beta-actin (B-actin) as loading control.

Biology Open • Supplementary information

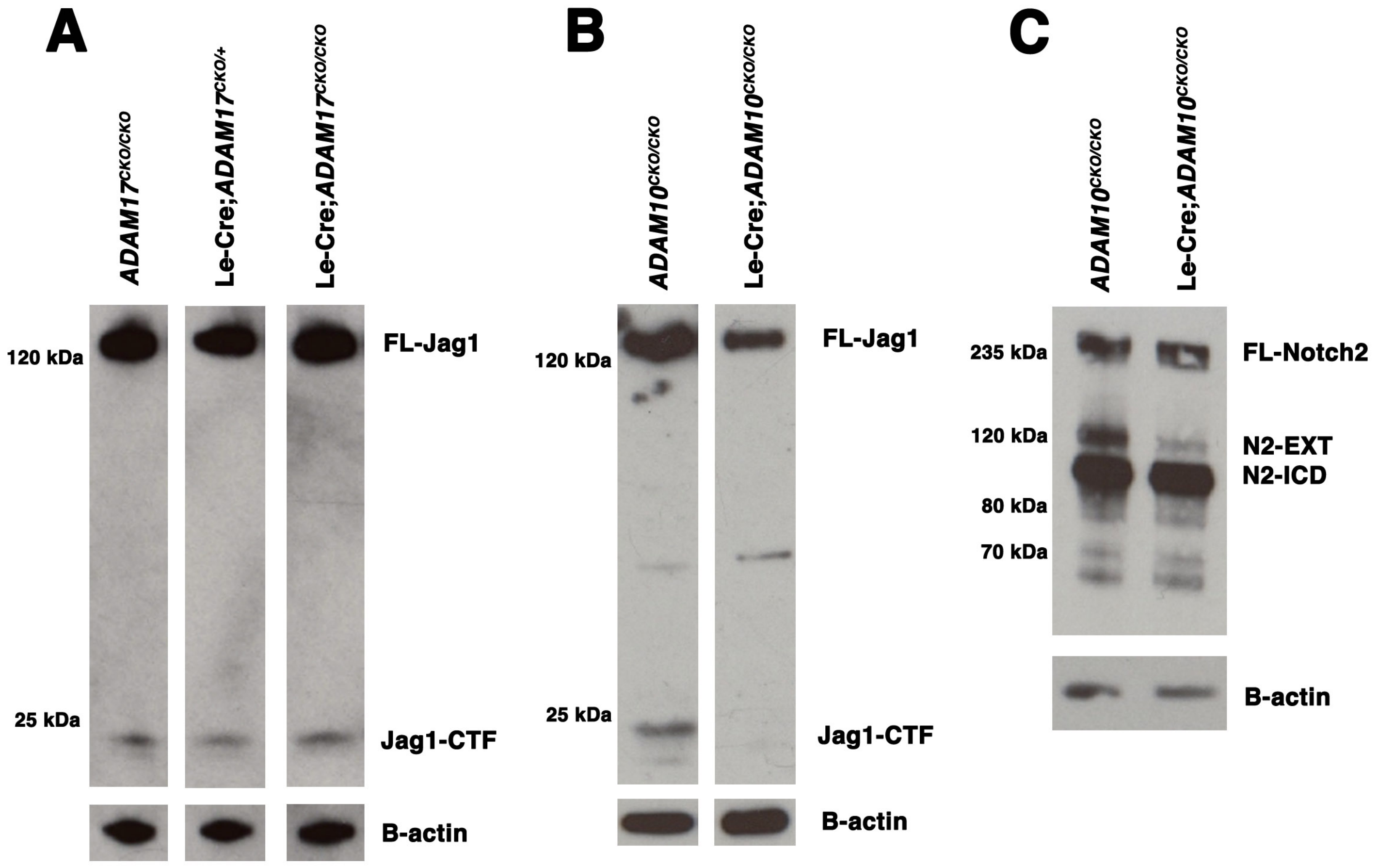

Figure S2. *ADAM10*, but not *ADAM17*, is necessary for Jag1-CTF production in the mouse lens. (A) Western blot analysis of lens protein lysates from E14.5 Le-Cre;*ADAM17* allelic series. Jag1-CTF generation is unaffected by removal of *ADAM17.* (B) Analogous Western blot assessment of Jag1 protein isoforms in lens protein lysates from E14.5 Le-Cre;*ADAM10CKO/CKO* embryos. There is an obvious loss of the Jag1-CTF after *ADAM10* removal. Blots in panels A,B probed with goat anti-Jag1 antibody (C) E14.5 Le-Cre;*ADAM10CKO/CKO* mutants also display a clear decrease in the extracellular-truncated form of the Notch2 receptor, N2-EXT. In all panels, anti Beta-actin reprobing (B-Actin) served as a loading control. n = 3 biologic replicates/genotype in A.
n= 1 biologic replicate/genotype in B,C, but independently performed (lysates loaded and probed by Western 3 times = technical replicates).

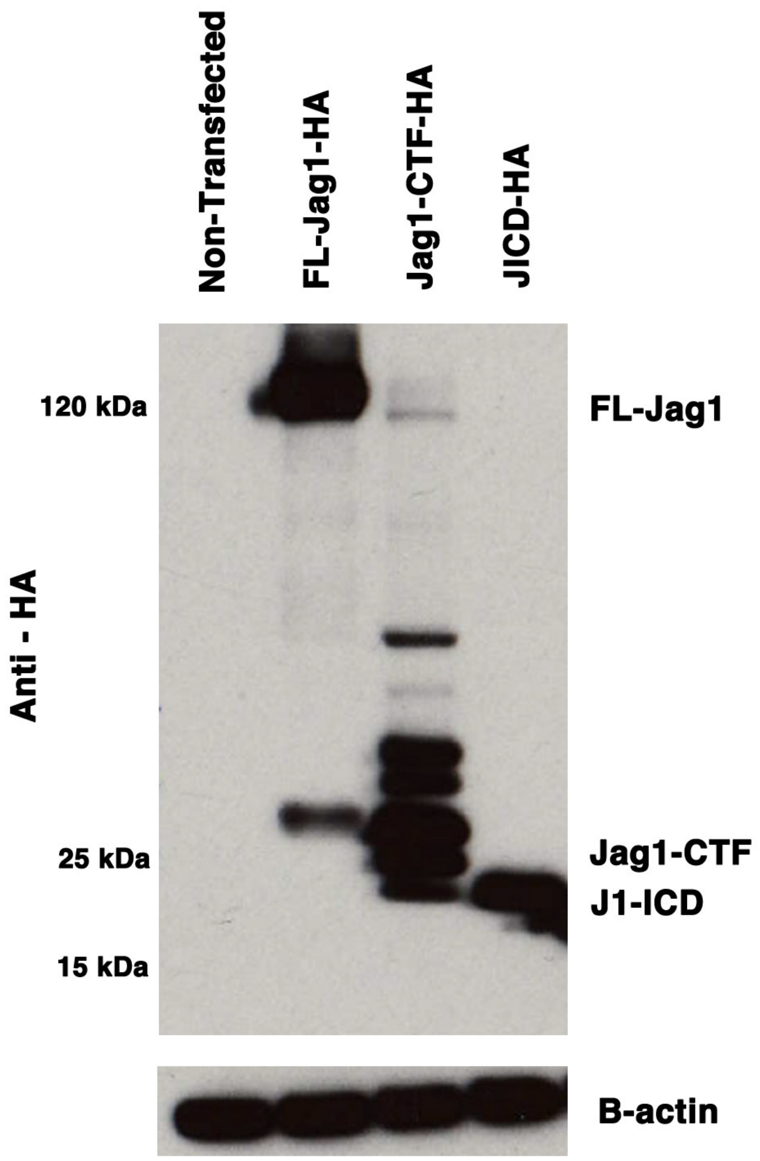

Figure S3. Anti-HA antibody Western blot.
B3 lens cells were transiently transfected with constructs for each rat epitope-tagged Jag1 isoform, then cell lysates harvested for analysis. Each lane was loaded with 50 ug of total protein. Non-transfected B4 lysate has no signal. B3 cell lysates after FL-Jag1-HA transfection contained Jag1-CTF-HA isoform, but not a J1-ICD-HA product. By contrast, B3 lysates from Jag1-CTF-HA transfections contained the J1-ICD-HA isoform, similar in size to in J1-ICD-HA after transfection of this isoform. Anti Beta-actin (B-actin) reprobing served as loading control. Blot is representative of n = 3 independent transfection to Western experiments.

Table S1. qPCR primer pairs obtained from the Harvard PrimerBank.

Gene	Forward primer 5'->3'	Reverse primer 3'->5'
β-*ACTIN*	CATGTACGTTGCTATCCAGGC	CTCCTTAATGTCACGCACGAT
ADAM10	ATGGGAGGTCAGTATGGGAATC	ACTGCTCTTTTGGCACGCT
ADAM17	GTGGATGGTAAAAACGAAAGCG	GGCTAGAACCCTAGAGTCAGG
JAG1	CAACCGTGCCAGTGACTATTTCTGC	TGTTCCCGTGAAGCCTTTGTTACAG
JAG2	AACGATACCACCCCGAATGAGG	GCTGCCACAGTAGTTCAGGTCTTTG
NOTCH1	GAGGCGTGGCAGACTATGC	CTTGTACTCCGTCAGCGTGA
NOTCH2	CAACCGCAATGGAGGCTATG	GCGAAGGCACAATCATCAATGTT
NOTCH3	CGTGGCTTCTTTCTACTGTGC	CGTTCACCGGATTTGTGTCAC
HES1	TCAACACGACACCGGATAAAC	GCCGCGAGCTATCTTTCTTCA
HES5	GATTCCTCTGTGTGGGTGGATG	GATTTTATTATGGCGGCTTCGG
PAX6	TTTGCCCGAGAAAGACTAGC	CATTTGGCCCTTCGATTAGA

Biology Open • Supplementary information
